# Does DNA Methylation of *PPARGC1A* Influence Insulin Action in First Degree Relatives of Patients with Type 2 Diabetes?

**DOI:** 10.1371/journal.pone.0058384

**Published:** 2013-03-07

**Authors:** Linn Gillberg, Stine Jacobsen, Rasmus Ribel-Madsen, Anette Prior Gjesing, Trine W. Boesgaard, Charlotte Ling, Oluf Pedersen, Torben Hansen, Allan Vaag

**Affiliations:** 1 Department of Endocrinology, Rigshospitalet, Copenhagen, Denmark; 2 Steno Diabetes Center, Gentofte, Denmark; 3 Section of Metabolic Genetics, The Novo Nordisk Foundation Center for Basic Metabolic Research, Faculty of Health Sciences, University of Copenhagen, Copenhagen, Denmark; 4 Hagedorn Research Institute, Gentofte, Denmark; 5 Department of Clinical Sciences, Lund University, Malmoe, Sweden; 6 Faculty of Health Sciences, University of Aarhus, Aarhus, Denmark; 7 Faculty of Health Sciences, University of Southern Denmark, Odense, Denmark; 8 Faculty of Health Sciences, University of Copenhagen, Copenhagen, Denmark; University of Tübingen, Germany

## Abstract

Epigenetics may play a role in the pathophysiology of type 2 diabetes (T2D), and increased DNA methylation of the metabolic master regulator peroxisome proliferator-activated receptor gamma coactivator 1 alpha (*PPARGC1A*) has been reported in muscle and pancreatic islets from T2D patients and in muscle from individuals at risk of T2D. This study aimed to investigate DNA promoter methylation and gene expression of *PPARGC1A* in skeletal muscle from first degree relatives (FDR) of T2D patients, and to determine the association with insulin action as well as the influence of family relation. We included 124 Danish FDR of T2D patients from 46 different families. Skeletal muscle biopsies were excised from *vastus lateralis* and insulin action was assessed by oral glucose tolerance tests. DNA methylation and mRNA expression levels were measured using bisulfite sequencing and quantitative real-time PCR, respectively. The average *PPARGC1A* methylation at four CpG sites situated 867-624 bp from the transcription start was associated with whole-body insulin sensitivity in a paradoxical positive manner (β = 0.12, *P* = 0.03), supported by a borderline significant inverse correlation with fasting insulin levels (β = −0.88, *P = *0.06). Excluding individuals with prediabetes and overt diabetes did not affect the overall result. DNA promoter methylation was not associated with *PPARGC1A* gene expression. The familiality estimate of *PPARGC1A* gene expression was high (*h^2^* = 79±27% (*h^2^*±SE), *P* = 0.002), suggesting genetic regulation to play a role. No significant effect of familiality on DNA methylation was found. Taken together, increased DNA methylation of the *PPARGC1A* promoter is unlikely to play a major causal role for the development of insulin resistance in FDR of patients with T2D.

## Introduction

Type 2 diabetes (T2D) is a multifactorial and slowly progressing multiple organ disease where metabolic abnormalities eventually leading to hyperglycemia are established long before the overt T2D diagnosis has become manifest [1]. Both genetic and non-genetic factors influence the development of T2D, and it has been estimated that the susceptibility to develop T2D in individuals with a T2D parent is 3–4 times higher compared to the background population [2]. Indeed, significant defects of peripheral (muscle) and hepatic insulin action, as well as of pancreatic beta cell function, have been reported to be present decades before first degree relatives (FDR) of patients with T2D are supposed to develop the disease [3], [4]. Genetic variants currently associated with susceptibility to T2D only explain up to 10% of the putative “primary” contribution to T2D risk [5] and it has therefore been debated whether the increased diabetes risk among FDR could be linked to epigenetic traits and not to classic genetic traits defined as alterations of the DNA sequence. However, little is known about the influence of heritability on epigenetic variation.

DNA methylation represents the most studied epigenetic trait and it is generally believed that increased methylation in the promoter region of tissue specific genes may confer transcriptional repression [6], [7]. This putative association has however been difficult to establish in several human studies [8]–[10]. Both DNA methylation and gene expression of the master metabolic regulator peroxisome proliferator-activated receptor gamma coactivator 1 alpha (*PPARGC1A*) has been extensively studied in relation to T2D. *PPARGC1A* upregulates transcription of genes involved in mitochondrial oxidative metabolism and biogenesis as well as skeletal muscle glucose transport [11], [12], and because mitochondrial defects have been associated with peripheral insulin resistance in healthy subjects [13], [14] it has been suggested that reduced *PPARGC1A* expression in skeletal muscle may be a primary feature of insulin resistance [11], [15]. Furthermore, *PPARGC1A* may be involved in biological functions with implications for in vivo insulin action including protection against oxidative stress, formation of muscle fiber types as well as regulation of microvascular flow [16], [17].

Reduced gene expression of *PPARGC1A* has been detected in skeletal muscle from both T2D patients [15], [18]–[20] and non-diabetic, insulin resistant FDR [21]. However, other studies did not consistently find decreased *PPARGC1A* expression in either T2D patients [22] or in healthy [22], [23] or insulin resistant [24] individuals with a family history of T2D.

In a study by Ling *et al.*, DNA methylation in a region of the *PPARGC1A* promoter located 867-624 base pairs (bp) upstream from the transcription start was higher in T2D patients, and furthermore showed a trend towards an inverse correlation with gene expression in pancreatic islets [25]. *PPARGC1A* gene expression as well as insulin secretion was reduced in T2D patients in this study, suggesting that DNA methylation in this distinct region of the promoter may influence the metabolic phenotype. Increased *PPARGC1A* promoter methylation has also been reported in skeletal muscle from individuals with impaired glucose tolerance (IGT) and T2D in a more proximal region of the *PPARGC1A* promoter located 337-37 bp upstream from the transcription start [18]. Collectively, increased methylation in the promoter region of *PPARGC1A* in individuals with T2D – or at increased risk of developing T2D – represent the hitherto most studied, and with some exceptions consistent, molecular epigenetic change potentially involved in the pathogenesis of insulin resistance and T2D.

To further investigate the role of DNA methylation in the development of T2D and insulin resistance, we examined *PPARGC1A* DNA promoter methylation in the two distinct regions previously examined and *PPARGC1A* gene expression in skeletal muscle from a unique population of 124 FDR of T2D patients from 46 different families. Furthermore, we determined the association between DNA methylation and clinical phenotypes including measures of insulin action. Finally, we evaluated whether DNA methylation and gene expression of *PPARGC1A* in muscle tissue demonstrate familial clustering and thus may be under genetic control.

## Materials and Methods

### Subjects

One-hundred-twenty-four Danish men and women from 46 different families were recruited in 2005–2007 as part of the EUGENE2 Consortium study population [26]. All individuals were FDR of patients with T2D and had either one parent with known T2D and the other parent with no family history of T2D and/or normal response to an oral glucose tolerance test (OGTT) (112 individuals), or a sibling (8 individuals) or child (4 individuals) with T2D at the time of recruitment. The common denominator of the study population was the known family history of T2D, and subjects were invited to participate irrespective of their glucose tolerance. The 46 families included 13 of size 1 (1 individual per family), 12 of size 2, 9 of size 3, 6 of size 4, 4 of size 5, 1 of size 6 and 1 of size 10. The study was approved by the Ethical Committee of Copenhagen and was in accordance with the principles of the Declaration of Helsinki II. All subjects signed an informed consent form prior to participation. Nine T2D patients received insulin treatment and were asked to discontinue the insulin treatment 12 hours prior to the clinical examination.

### Clinical Examinations

Participants were examined by anthropometric measurements, and a standard OGTT was performed. Blood samples for measurements of plasma glucose and serum insulin were drawn every 30 min during the OGTT until 180 min after ingestion of the 75 g glucose solution. On another occasion after an overnight fast, skeletal muscle tissue was excised from the *vastus lateralis* muscle using a Bergström needle and snap frozen in liquid nitrogen at −80°C. Plasma glucose and serum insulin levels were measured as previously described [26]. Insulin sensitivity was estimated from plasma glucose and serum insulin levels obtained in the fasted state and during the OGTT, calculating the homeostatic model assessment of insulin resistance (HOMA-IR) [27] as well as the whole-body Matsuda insulin sensitivity index (ISI) [28]. Estimates of insulin secretion was reported as HOMA of β-cell function (HOMA-β) [27] and the corrected insulin response (CIR) calculated as (serum insulin_30 min_ [pmol l^−1^]×0.144×100)/(plasma glucose_30 min_ [mmol l^−1^]×(plasma glucose_30 min_ [mmol l^−1^] –3.89)) [29].

### DNA Methylation

**Figure 1:**
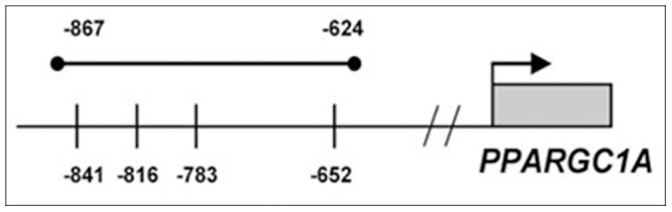
CpG sites analyzed in the *PPARGC1A* promoter. The CpG sites investigated are marked with a perpendicular line.

Genomic DNA was isolated from muscle biopsies using the DNeasy Blood and Tissue Kit (Qiagen, Hilden, Germany). Bisulfite conversion of 500 ng DNA was performed using the EZ DNA Methylation-Gold Kit (Zymo Research, Orange, CA, USA). Site-specific DNA methylation at four CpG sites in a subpart of the *PPARGC1A* promoter located 867-624 bp upstream from the transcription start (Figure 1) was determined by bisulfite sequencing [30]. These CpG sites are identical to the CpG sites investigated by Ling *et al.* where the numbering of the CpG sites (−961, −936, −903, −772) was carried out based on the translational start site situated 120 bp from the transcriptional start site. The bisulfite treated DNA was amplified with forward primer 5′-TATTTTAAGGTAGTTAGGGAGGAAA-3′ and reverse primer 5′-CCCATAACAATAAAAAATACCAACTC-3′ designed by MethPrimer [31]. PCR amplicons were verified by electrophoresis through a 3% ethidium bromide stained agarose gel and treated with ExoSAP-IT (USB Corp, Cleveland, OH, USA) to remove small contaminating fragments. Sequencing PCR was performed using the BigDye Terminator v3.1 Cycle Sequencing Kit (Applied Biosystems, Foster City, CA, USA). DNA samples were precipitated with the BigDye XTerminator Purification Kit (Applied Biosystems), and the samples were sequenced in an ABI 3130xl Genetic Analyzer (Applied Biosystems). The sequence trace files were subjected to quality control and methylation quantification using the epigenetic sequencing methylation (ESME) analysis software version 3.2.1 (Epigenomics, Berlin, Germany). For each sample, the sequence regularity was checked manually by visualization of the ESME output picture files and exclusion of data among repeated measurements was set to a cut-off of 10% for the largest methylation difference among the triplicate measurements. DNA methylation at four CpG sites in the proximal *PPARGC1A* promoter (−260, −136, −99, −94) was analyzed with pyrosequencing. Two primer assays covering the CpG of interest were designed using the PyroMark Assay Design 2.0 software (Qiagen). The PyroMark PCR kit (Qiagen) was used for amplifying bisulfite converted DNA according to manufacturer’s protocol. The PyroMark Q96 Vacuum Workstation was used for preparing the samples and pyrosequencing was performed with the PyroMark Q96 ID instrument (Qiagen). Data were analyzed with the Pyrogram software v.2.5.8. Pyrograms for all samples were checked manually to validate the quality of the sequencing analysis and samples with unreliable methylation results based on warning messages given by the software (uncertainties due to baseline shift, low signal to-noise ratio, low peak height and high peak-height deviation at positions close to the CpG site analyzed) were re-run. DNA methylation results that were subjected to these warning messages after the re-analysis were excluded.

### Gene Expression

Total RNA was extracted from muscle tissue using TRI Reagent (Sigma-Aldrich, St. Louis, MO, USA) and converted to cDNA by use of the QuantiTect Reverse Transcription Kit (Qiagen). Gene expression was determined by quantitative real-time PCR with the ABI PRISM 7900HT Sequence Detection System (Applied Biosystems) using gene-specific primers and TaqMan probes for *PPARGC1A* (Hs00173304_m1) (Applied Biosystems). Each sample was measured in duplicate, and the standard curve approach was used for quantification. *PPARGC1A* mRNA samples with Ct values above 31 cycles or a Ct difference above 0.35 on repeated measurements were re-run. Samples that exceeded these cut-offs on the re-analysis were excluded. The *PPARGC1A* mRNA quantity was normalized to the relative amount of cDNA content in each sample as measured in triplicates with the Quant-iT OliGreen ssDNA Assay Kit (Invitrogen, Carlsbad, USA) in combination with the ABI PRISM 7900HT Sequence Detection System [32], [33]. This method of normalization is based on the Oligreen dye, which binds with a preferential affinity to ssDNA and upon binding emits fluorescence that can be measured in a single step during adapted thermal conditions. The cDNA content of each sample was calculated after being plotted as a function of the cDNA concentration (i.e. to the linearity of a cDNA standard curve). Samples with a standard deviation divided by average of above 10% on triplicate measurements were re-run, and samples exceeding the cut-off of 10% on repeated measurements after the re-analysis were excluded. Also, *PPARGC1A* mRNA was normalized to cyclophilin (*PPIA*) gene expression of each sample (n = 105), but due to significant associations between *PPIA* mRNA expression and both age (*P* = 0.04) and gender (*P* = 0.002), the *PPARGC1A* mRNA normalized to the cDNA content was considered to be a more robust method for normalization.

### Statistical Analysis

Quantitative data were analyzed with linear mixed models in RGui version 2.13.1 (http://www.r-project.org). Residuals from the mixed model analyses were checked for normality by qq-plots. Given that all participants included are of very high risk of developing T2D, and the fact that definition of prediabetic and overt diabetic status is based on arbitrary criteria, we analyzed the results of the study considering the total population of participants’ altogether. In order to test the robustness of our findings, and due to the heterogeneous study population, we subsequently analyzed all results for the non-diabetic and the T2D subjects separately. All analyzes were adjusted for age, gender, body mass index (BMI) and glucose tolerance status (fixed effects). Pedigree (coded as a family number) was included as a random factor. Results from the mixed models are presented as effect estimates (β) with 95% confidence intervals and *P*-values. Unpaired, non-parametric tests and Spearman’s correlation tests (ρ) were performed in SAS 9.1 (SAS Institute Inc., Cary, NC). *P*-values ≤0.05 were considered significant in two-tailed testing. *PPARGC1A* DNA methylation and gene expression was analyzed in skeletal muscle biopsies from all 124 participants and reliable experimental data was obtained for 117 individuals (DNA methylation, region −867 to −624), 109 individuals (DNA methylation, CpG −260) and 102 individuals (gene expression) respectively. *PPARGC1A* DNA methylation of CpG −136, −99 and −94 was analyzed and obtained from 15 individuals. Because only one subject had a methylation percentage above 0% on one of the CpG sites (NGT subject, 3.87% on CpG −99), and the remaining 14 subjects had no (0%) methylation on all 3 CpG sites, we decided not to analyze methylation of these CpG sites in the remaining samples.

The influence of the familial relation on methylation and gene expression of *PPARGC1A* was estimated from a polygenic model as the proportion of the additive genetic variation on the total variation (variance component approach). The term familiality is used instead of (narrow sense) heritability to emphasize that the resulting estimate not only provides information about genetic similarity, but also shared environmental effects in the FDR group. The familiality of DNA methylation and gene expression was adjusted for age, gender, BMI and glucose tolerance status using the SOLAR software (http://solar.txbiomedgenetics.org).

## Results

### Subjects Characteristics

The study population consisted of 45 men and 79 women being 32–83 years old (Table 1). The group had varying degrees of BMI (18–47 kg/m^2^) and 45 individuals were obese (BMI above 30 kg/m^2^). According to the 1999 WHO diagnostic criteria, 80 individuals had normal glucose tolerance (NGT), 5 impaired fasting glucose (IFG), 12 IGT, 2 had both IFG and IGT and 25 individuals had T2D. In this study, the 19 individuals with IFG and/or IGT were grouped together. Individuals in the IFG/IGT subgroup had significantly higher age, fasting triglycerides, plasma glucose and serum insulin levels compared to the NGT subgroup, and were significantly more insulin resistant based on Matsuda ISI (Table 1). Individuals with T2D were characterized by higher age, BMI, HbA1C, fasting serum insulin and plasma triglyceride levels, and lower insulin sensitivity (Matsuda ISI) and insulin secretion (HOMA-β and CIR) compared to NGT subjects. Furthermore, they showed significantly higher HbA1C and lower 2 h OGTT related serum insulin and insulin secretion compared to IFG/IGT subjects. All subgroups differed in fasting and 2 h OGTT-related plasma glucose levels and in estimates of in vivo insulin resistance by HOMA-IR (Table 1). Insulin sensitivity based on Matsuda ISI was significantly lower in men than in women (6.3 vs. 8.7).

**Table 1:** Clinical characteristics of the FDR group (n = 124) stratified according to glucose tolerance status.

	NGT	IFG/IGT	T2D
*n (total)*	*80*	*19*	*25*
*n (men/women)*	*23/57*	*11/8*	*11/14*
Age (years)	51.5±10.0	54.8±10.7*	60.4±10.3^++^
BMI (kg/m^2^)	27.2±5.2	29.8±5.4	31.3±4.7^++^
HbA1C (%)	5.3±0.3	5.4±0.4	7.3±1.6^++,§§^
**Fasting**			
Plasma glucose (mmol l^−1^)	5.4±0.4	5.8±0.5**	9.9±4.0^++,§§^
Serum insulin (pmol l^−1^)	41.7±33.7	60.2±40.1*	64.9±50.6^++^
Plasma triglyceride (mmol l^−1^)	1.2±0.6	1.7±0.7**	2.0±1.4^+^
**2h OGTT related**			
Plasma glucose (mmol l^−1^)	5.8±1.1	8.1±1.7**	16.4±5.0^++,§§^
Serum insulin (pmol l^−1^)	215±196	497±465*	257±318^§^
**Insulin sensitivity**			
HOMA-IR	1.5±1.3	2.2±1.5*	4.2±4.2^++,§^
Matsuda ISI	9.5±5.1	5.4±3.3**	4.3±2.1^++^
**Insulin secretion**			
HOMA-β	63.9±44.5	77.5±53.4	38.4±30.2^++,§^
CIR	8.6±5.4	6.1±4.0	1.6±1.9^++,§§^

Data are mean ± SD. Significant differences between NGT and IFG/IGT at *P<0.05. **P<0.001. Significant differences between NGT and T2D at ^+^ *P*<0.05, ^++^ *P*<0.001. Significant differences between IFG/IGT and T2D at ^§^ *P*<0.05, ^§§^ *P*<0.001. All parameters except age and BMI were analyzed with unpaired non-parametric tests due to lack of normal distribution. Indices of insulin sensitivity and insulin secretion were calculated as described in subjects and methods. BMI, body mass index; CIR, corrected insulin response; HOMA-β, homeostatic model assessment of β-cell function; HOMA-IR, homeostatic model assessment of insulin resistance; IFG, impaired fasting glycemia; IGT, impaired glucose tolerance; NGT, normal glucose tolerance; OGTT, oral glucose tolerance test; T2D, type 2 diabetes.

### *PPARGC1A* mRNA Expression

**Figure 2:**
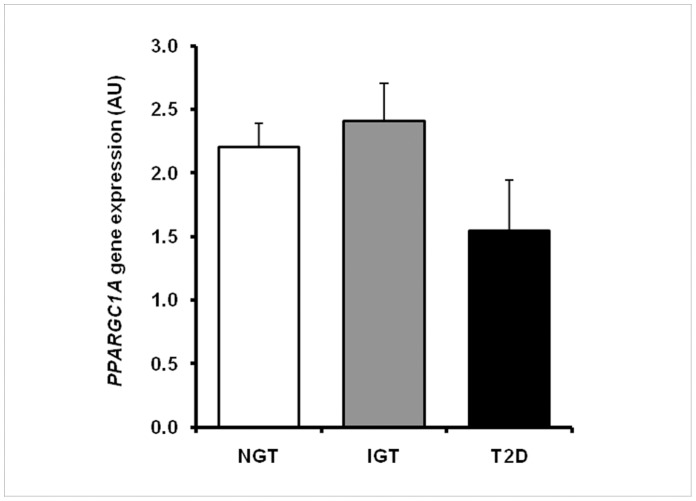
Skeletal muscle gene expression of *PPARGC1A*. Participants are stratified according to glucose tolerance status (NGT, IFG/IGT, T2D). Data are mean±SE.

Skeletal muscle gene expression of *PPARGC1A* was not significantly different between the NGT, IFG/IGT and T2D subgroups (Figure 2). *PPARGC1A* gene expression did not show any significant associations with age, gender, BMI, fasting glucose, insulin or triglyceride levels, or with insulin sensitivity based on HOMA-IR or Matsuda ISI indices in the whole FDR group (data not shown). When the FDR group was divided into non-diabetic and T2D subgroups, *PPARGC1A* gene expression in the T2D subgroup (n = 25) was significantly positively correlated with whole-body insulin sensitivity (Matsuda ISI) (β = 0.16 (0.03;0.29) *P* = 0.02). Similar results of *PPARGC1A* gene expression was obtained when *PPARGC1A* mRNA was normalized to *PPIA* mRNA expression as compared to cDNA content (data not shown).

### *PPARGC1A* DNA Methylation

**Figure 3:**
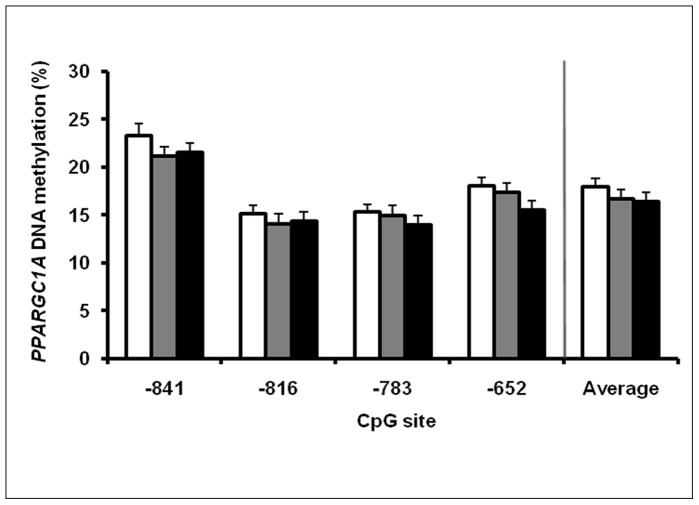
DNA methylation at different CpG sites in the promoter region of *PPARGC1A* in skeletal muscle. The FDR participants are stratified according to glucose tolerance status: NGT (*white bars*), IFG/IGT (*grey bars*), T2D (*black bars*). Data are mean ± SE.

**Figure 4:**
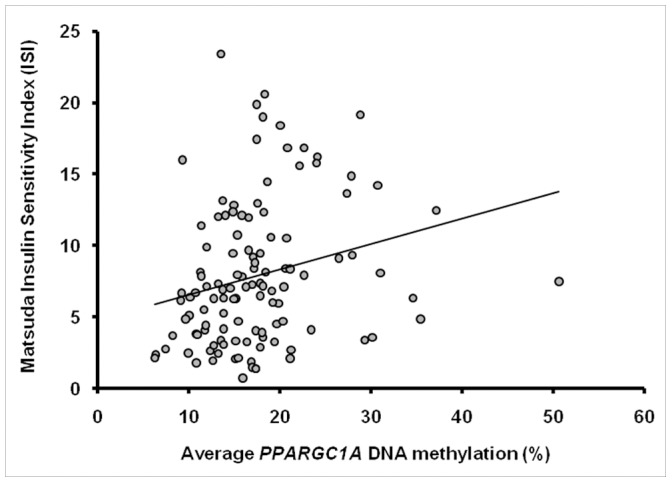
Correlation between average skeletal muscle DNA methylation of the *PPARGC1A* promoter and whole-body insulin sensitivity. Methylation is shown in percentage and Matsuda ISI was used as a marker of whole-body insulin sensitivity in the FDR group. β = 0.12 (0.01;0.24) *P* = 0.03, adjusted for age, gender, BMI, glucose tolerance and family pedigree. Unadjusted correlation (Spearman’s correlation): ρ 0.31, *P* = 0.0007.

**Figure 5:**
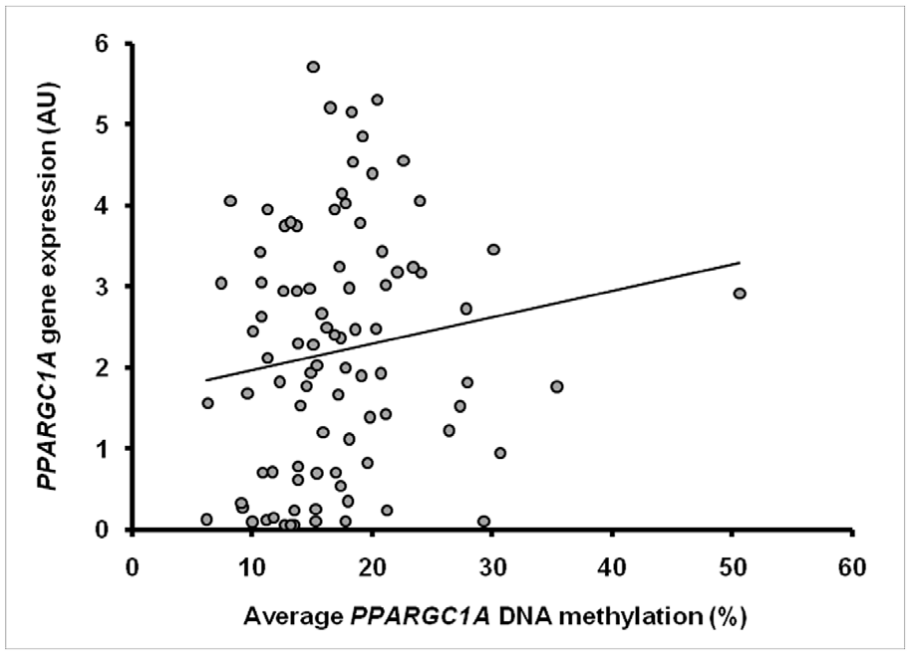
Correlation between average skeletal muscle DNA methylation and gene expression of *PPARGC1A*. Methylation is show in percentage and gene expression in arbitrary units (AU). β = 0.013 (−0.034;0.059), *P = *0.59, adjusted for age, gender, BMI, glucose tolerance and family pedigree. Unadjusted (Spearman’s correlation): ρ 0.22, *P* = 0.04.methylation at CpG site −260 in the *PPARGC1A* promoter was 0% in 98 individuals and 4–10% in 11 individuals (4 with T2D and 7 with NGT). The DNA methylation at CpG site −260 was not different among T2D (1.1±1.7) compared to NGT subjects (0.74±2.3), and there were no significant associations between DNA methylation and whole body insulin sensitivity, gene expression or any other clinical parameter (data not shown).

*PPARGC1A* DNA methylation in the region 867-624 bp upstream from the transcription start was not affected by gender, age, BMI, fasting plasma glucose or triglyceride levels, or with insulin resistance based on the HOMA-IR index. *PPARGC1A* methylation did not differ significantly between the NGT, IFG/IGT and T2D subgroups (Figure 3). Interestingly, the average degree of *PPARGC1A* DNA methylation for the whole FDR group was positively associated with whole-body insulin sensitivity (Matsuda ISI) (β = 0.12 (0.01;0.24) *P* = 0.03) (Figure 4). The positive correlation with insulin action was significant on one (site −783: β = 0.12 (0.002;0.23) *P* = 0.05), and borderline significant on two (site −841: β = 0.08 (−0.004;0.16) *P* = 0.06; site −816: β = 0.09 (−0.02;0.20) *P* = 0.09) out of the four CpG sites. This finding was also supported by a borderline significant inverse correlation between average DNA methylation and fasting serum insulin levels (β = −0.88 (−1.80;0.04) *P = *0.06). When subjects were divided according to glucose tolerance status, the relation between average *PPARGC1A* methylation and whole-body insulin sensitivity remained significant in the NGT (β = 0.14 (0.006;0.28) *P* = 0.04) and the NGT/IFG/IGT (non-diabetic) group (β = 0.14 (0.02;0.27) *P* = 0.02), but not in the IFG/IGT or T2D group. Separate analyses of non-diabetic subjects (n = 99) also revealed significant inverse correlations between average *PPARGC1A* DNA methylation and fasting insulin (β = −0.83 (−1.64;−0.02) *P* = 0.05), fasting glucose (β = −3.86 (−7.80;0.07) *P* = 0.05) and insulin resistance based on HOMA-IR (β = −1.65 (−3.02;−0.27) *P* = 0.02). Finally, *PPARGC1A* promoter methylation in the region 867–624 bp upstream from the transcription start did not show a significant inverse correlation with *PPARGC1A* gene expression (Figure 5).

### Familiality of *PPARGC1A* DNA Methylation and Gene Expression

The familiality for *PPARGC1A* gene expression (*h^2^* = 79±27%, *P* = 0.002) (*h^2^*±SE) was higher than for *PPARGC1A* promoter methylation (*h^2^* = 16±17%, *P* = 0.16), and only the gene expression showed a statistically significant influence by familiality.

## Discussion

Several studies have suggested that skeletal muscle DNA methylation and gene expression of *PPARGC1A* may be involved in the pathogenesis of T2D [8], [18], [20], [21], [25]. Our current study including 124 Danish FDR of T2D patients did however not show any association between glucose tolerance status and *PPARGC1A* promoter methylation or gene expression in skeletal muscle. Unexpectedly, and opposite to our *a priori* hypothesis, the degree of *PPARGC1A* methylation correlated positively with whole-body insulin sensitivity, and inversely with fasting insulin levels. Our data furthermore indicate that genetics and/or shared environmental effects play a role in the regulation of *PPARGC1A* gene expression, whereas *PPARGC1A* DNA methylation seems less influenced by familiality factors. Overall, our data do not support the view that skeletal muscle *PPARGC1A* promoter methylation plays any major causal role in the pathogenesis of T2D, at least not among individuals with a family history of T2D.

Skeletal muscle gene expression of *PPARGC1A* and genes involved in oxidative phosphorylation has been extensively studied in relation to prediabetes and T2D, and increased skeletal muscle expression of *PPARGC1A* is believed to contribute to improved insulin sensitivity [15], [34], [35]. We were unable to establish an association between *PPARGC1A* gene expression and in vivo insulin resistance in the total population of FDR in our study. However, when analyzed according to glucose tolerance status, *PPARGC1A* gene expression in the T2D subgroup showed a significant positive correlation with whole-body insulin sensitivity, which was in accordance with our hypothesis.

The FDR subjects studied have increased risk of T2D due to having a parent, sibling or child diagnosed with the disease. Also the participants are subdivided into 46 different families, which makes our group more genetically homogenous compared to the general Danish population [26]. It could be argued that we lack a control group of individuals without a family history of T2D, making our results more difficult to contrast with some of the previous studies. However, in three previous studies the muscle *PPARGC1A* gene expression in healthy or insulin resistant individuals with a family history of T2D was similar to healthy matched controls without a family history of T2D [22]–[24].

The association between insulin sensitivity and *PPARGC1A* gene expression in skeletal muscle has been investigated in previous studies with conflicting results [21], [24], [36]. Patti *et al.* found reduced expression of *PPARGC1A* in insulin resistant compared to insulin sensitive offspring of parents with T2D [21], whereas Morino *et al.* found no alteration in gene expression or protein content of *PPARGC1A* in young, insulin resistant offspring of T2D parents compared to controls [24]. Also, T2D has been associated with a reduced muscle *PPARGC1A* gene expression in some [18]–[21], but not all [22] studies. In accordance with our results, Palsgaard *et al.* found similar *PPARGC1A* gene expression in T2D compared to normoglycemic subjects [22]. Collectively, the present study together with previous studies suggests that the expected inverse relationship between skeletal muscle *PPARGC1A* gene expression and insulin resistance or T2D is not a consistent and reproducible finding. Interestingly, intervention studies have shown that skeletal muscle gene expression of *PPARGC1A* was downregulated in young healthy men after 9 days of bed rest [37], and after a 5 day high-fat high-calorie diet in low birth weight men with an increased risk of T2D [8]. Moreover, the increase in *PPARGC1A* mRNA and protein content following exercise is reduced and delayed in muscle from insulin resistant subjects [38]. Therefore, a metabolic challenge could be necessary to unmask the association between *PPARGC1A* expression and prediabetes in the FDR subjects included in this study.

DNA promoter methylation could be one among several mechanisms regulating *PPARGC1A* gene transcription in not only skeletal muscle, but also in other primary diabetogenic tissues such as the pancreatic beta cell, liver or adipose tissue. Only muscle was addressed in this study, and indeed *PPARGC1A* DNA methylation and/or gene expression could be more tightly linked and functionally important with respect to T2D pathogenesis in tissues other than muscle. Our recent study of skeletal muscle and subcutaneous adipose tissue from monozygotic twins showed that between these different tissues involved in peripheral glucose metabolism, numerous DNA methylation differences were found. However, between twin differences of DNA methylation in each specific tissue were modest [39]. These findings emphasize the robustness of the tissue specific DNA methylation patterns.

Importantly, the CpG sites analyzed in the *PPARGC1A* promoter were carefully selected based on previous studies where DNA methylation of these sites in skeletal muscle and pancreatic islets show signs of metabolic relevance [8], [18], [25], [37]. We were however unable to establish this relationship in skeletal muscle from the FDR group despite the large number of participants. In spite of a fairly convincing belief among many that promoter DNA methylation causes transcriptional repression, poor correlations between these factors have been reported for most genes in large-scale studies such as the Human Epigenome Project, where one third of the differentially methylated promoter regions were found to correlate inversely with gene transcription [9]. Possible explanations of the lack of an inverse correlation between DNA promoter methylation and gene expression of *PPARGC1A* in our study may be that the amount of *PPARGC1A* mRNA analyzed by quantitative real-time PCR may not reflect *PPARGC1A* transcription in cases where the mRNA turnover rate is high. To this end, associations between *PPARGC1A* mRNA and promoter methylation might only be unmasked when the regulation is activated in response to metabolically challenged states, as for example during exposure to a diet rich in calories and/or after high-intense physical exercise. Cyclical, rapid changes in methylation status at promoter CpG dinucleotides of transcriptionally active genes may constitute another explanation of the lack of correlation with gene expression, as previously shown in the promoter of estrogen receptor alpha [40]. Finally, we cannot exclude the possibility that other CpG sites in the *PPARGC1A* promoter may play a more important role in the regulation of gene expression and subsequent metabolic actions.

In a study by Barres *et al.* DNA methylation of non-CpG sites in a region −337 to −37 bp upstream from the transcription start was increased in skeletal muscle from both IGT and T2D compared to NGT subjects [18]. The promoter methylation was inversely correlated with gene expression and consequently suggested to be associated with impaired insulin sensitivity. Therefore, it was unexpected for us to observe that increased DNA methylation of CpG sites in the region −867 to −624 bp upstream from the transcription start was associated with increased (and not decreased) whole-body insulin sensitivity, supported by a borderline significant inverse association with insulin levels in the fasting state. Excluding individuals with glucose intolerance and/or overt diabetes did not affect the significance of these associations.

In this study, we focused primarily on the four methylation sites that we previously found to exhibit increased methylation in prediabetic and T2D subjects [8], [25]. In order to address to potential impact of DNA methylation at the sites closer to transcription start, we measured these too in the present cohort. However, we found these sites to be without any detectable DNA methylation in the majority of the study subjects. Accordingly, we were unable to find any differences between groups and we furthermore could not determine any relationship with gene expression or insulin action.

Whole-body insulin sensitivity as estimated by the Matsuda ISI reflects insulin sensitivity in both liver and peripheral tissues, and has been shown to correlate with peripheral insulin sensitivity as measured by the gold standard hyperinsulinemic euglycemic clamp technique [28]. A possible explanation for the paradoxical association between increased *PPARGC1A* DNA methylation and improved insulin sensitivity, as well as reduced insulin levels, in the FDR group could be a counter-regulatory cellular mechanism. One could imagine that *de novo* methylation processes may become activated in FDR with an improved insulin sensitivity state of the body, such that the *PPARGC1A* promoter region is methylated to shut off pathways activated by *PPARGC1A*, all together balancing the regulation of the system by a feedback mechanism. Also, other studies by our group have shown that *PPARGC1A* promoter methylation at the identical CpG sites in muscle tissue from young, healthy men is sensitive to physiological and metabolic challenges such as 5-days high-fat high-calorie diet [8] and 9 days of bed rest [37]. Whatever the mechanism may be, the hypothesis that increased methylation of the *PPARGC1A* promoter is associated with insulin resistance seems questionable and needs further investigation.

The inclusion of families in the FDR group allowed us to estimate the influence of familiality (i.e. genetic effects and shared environmental effects) on DNA methylation and gene expression of *PPARGC1A*. Our data clearly demonstrate that *PPARGC1A* gene expression was highly influenced by familiality (*h^2^* = 79±27%, *P* = 0.002), whereas the influence of familiality on *PPARGC1A* promoter methylation was low and insignificant (*h^2^* = 16±17%, *P* = 0.16). We believe that polymorphisms either within, adjacent to or distant from the regulatory region and the gene body may be able to modulate *PPARGC1A* transcription and/or *PPARGC1A* mRNA degradation. Also, heritable effects of DNA methylation on other CpG sites than the ones investigated in this study could also possibly be involved in the regulation of *PPARGC1A* transcription. The influence of heritability on skeletal muscle *PPARGC1A* gene expression was previously investigated in young and elderly monozygotic and dizygotic twins and although the heritable effect was not statistically significant in either young or elderly twins, a polymorphism in *PPARGC1A*, the Gly482Ser variant, was associated with *PPARGC1A* gene expression [41]. Conversely, a genome-wide analysis of gene expression in lymphoblastoid cell lines from monozygotic twin pairs suggested a significant heritable component of gene expression levels with an average broad-sense heritability estimated to 31% [42]. Heritable effects of DNA methylation was recently investigated at 1760 CpG sites in 186 regions in the human major histocompatibility complex in CD4+ lymphocytes from 49 monozygotic and 40 dizygotic Norwegian twin pairs [43]. In accordance with our data, they reported low heritability estimates for DNA methylation, ranging from 2% to 16% across four types of gene regions in the major histocompatibility complex. The limited genetic contribution to *PPARGC1A* DNA methylation variation in skeletal muscle from our group, together with our previous studies of *PPARGC1A* methylation in relation to physiological and dietary challenges [8], [37], all together suggests that the CpG sites that we have investigated in the *PPARGC1A* promoter have a highly dynamic and relatively fast regulation of methylation that mainly is controlled by environmental effects such as the physiological/metabolic state of the body.

In conclusion, a paradoxical positive relationship between *PPARGC1A* DNA methylation and insulin action in skeletal muscle was demonstrated. These data challenge the notion of an adverse effect of *PPARGC1A* DNA methylation on insulin action, at least among individuals with a family history of T2D. Furthermore, data from the FDR group revealed a significant effect of familiality on *PPARGC1A* gene expression contrasting the absence of any significant familiality on degree of DNA methylation. Further studies are needed to increase our understanding of the impact of DNA methylation and gene expression of *PPARGC1A* as well as other candidate genes on the pathogenesis of T2D.
