# Correction: Does DNA Methylation of *PPARGC1A* Influence Insulin Action in First Degree Relatives of Patients with Type 2 Diabetes?

**DOI:** 10.1371/annotation/5c3cf392-57b5-4e80-9a66-4997d10200ae

**Published:** 2013-06-21

**Authors:** Linn Gillberg, Stine Jacobsen, Rasmus Ribel-Madsen, Anette Prior Gjesing, Trine W. Boesgaard, Charlotte Ling, Oluf Pedersen, Torben Hansen, Allan Vaag

The name of the second author is misspelled. The correct name is: Stine C. Jacobsen. The correct citation is: Gillberg L, Jacobsen SC, Ribel-Madsen R, Gjesing AP, Boesgaard TW, et al. (2013) Does DNA Methylation of *PPARGC1A* Influence Insulin Action in First Degree Relatives of Patients with Type 2 Diabetes? PLoS ONE 8(3): e58384. doi:10.1371/journal.pone.0058384. The correct abbreviation in the Author Contributions section is: SCJ.

In addition, a sentence was incorrectly placed in the legend of Figure 5. The sentence is as follows: "DNA methylation at CpG site -260 in the *PPARGC1A* promoter was 0% in 98 individuals and 4-10% in 11 individuals (4 with T2D and 7 with NGT). The DNA methylation at CpG site -260 was not different among T2D (1.1±1.7) compared to NGT subjects (0.74±2.3), and there were no significant associations between DNA methylation and whole body insulin sensitivity, gene expression or any other clinical parameter (data not shown)." This sentence should be read as the final sentence in the "*PPARGC1A* DNA Methylation" section of Results. 

